# Imaging characteristics of slow-growing soft tissue chondroma of the tongue: A case report

**DOI:** 10.1097/MD.0000000000029276

**Published:** 2022-05-20

**Authors:** Yoonsoo Park, Ha Youn Kim, In-kyu Yu, Dong-Sik Jang, Joo Heon Kim

**Affiliations:** a Department of Radiology, Eulji University Hospital, Eulji University College of Medicine, Daejeon, Korea; b Department of Otorhinolaryngology, Eulji University Hospital, Eulji University College of Medicine, Daejeon, Korea; c Department of Pathology, Eulji University Hospital, Eulji University College of Medicine, Daejeon, Korea.

**Keywords:** CT, MRI, slow-growing tongue mass, soft tissue chondroma

## Abstract

**Introduction:**

Extraskeletal soft tissue chondroma (STC) is a rare benign tumor. Soft-tissue chondromas rarely occur in the oral cavity. In this study, we aimed to confirm a slow-growing tongue mass using magnetic resonance imaging.

**Patient concerns:**

A 60-year-old woman presented with a painful, slow-growing tongue mass that had persisted for 17 years. Intraoral examination revealed a pedunculated mass covered with mucosa on the right side of her tongue.

**Diagnosis:**

CT and MRI revealed a lobulated heterogeneously enhancing mass without calcification. Compared with previous images obtained 17 years prior, the mass presented slow growth, more prominent enhancement, and lobulated contour. Histopathological examination confirmed the presence of STC.

**Interventions:**

Excision of the mass surrounding normal tissue was performed under general anesthesia.

**Outcomes:**

During 1-year follow-up period, no recurrence was observed.

**Conclusions:**

In this study, STC lesions were slow-growing, and changed from weakly homogeneous enhancement and clean margins to markedly heterogeneous enhancement and lobulated margins over time.

## 1 Introduction

Soft tissue chondromas (STCs) are rare, benign tumors that occur in extraosseous and extrasynovial locations.^[1]^ STCs usually occur in the soft tissues of the hands and feet, and fingers are frequently affected.^[2]^ Other locations include the dura, larynx, skin, and fallopian tubes.^[3]^ STCs rarely occur in the oral cavity.^[1]^ The subsites where these tumors have been reported include the tongue, cheek, hard and soft palate, masseter muscle, and masticatory space.^[4]^

STCs usually present as asymptomatic, slow-growing, well-defined nodules that expand into surrounding tissues. They are predominantly composed of adult-type hyaline cartilage, devoid of other differentiated elements, except for osseous, fibrous, and/or myxoid stroma.^[3]^

The tongue is the most common site of occurrence of oral STCs. Oral STC presents as an elastic semicircular nodule with a diameter of approximately 5 mm on the dorsum of the tongue. Forty-five cases of fibrochondroma of the tongue have been reported in literature.^[1]^

However, there have been few studies on the imaging findings of chondromas in the oral cavity. Therefore, in this study, slow-growing chondrocytes were identified using computed tomography (CT) and magnetic resonance imaging (MRI), and the changes in radiological findings as they grew were investigated.

## 2 Case presentation

A 60-year-old woman presented with complaints of a slightly painful, slow-growing, 17-year standing lesion. Intraoral examination revealed a pedunculated multilobulated mass covered with mucosa on the right side of the tongue.

**Figure 1:**
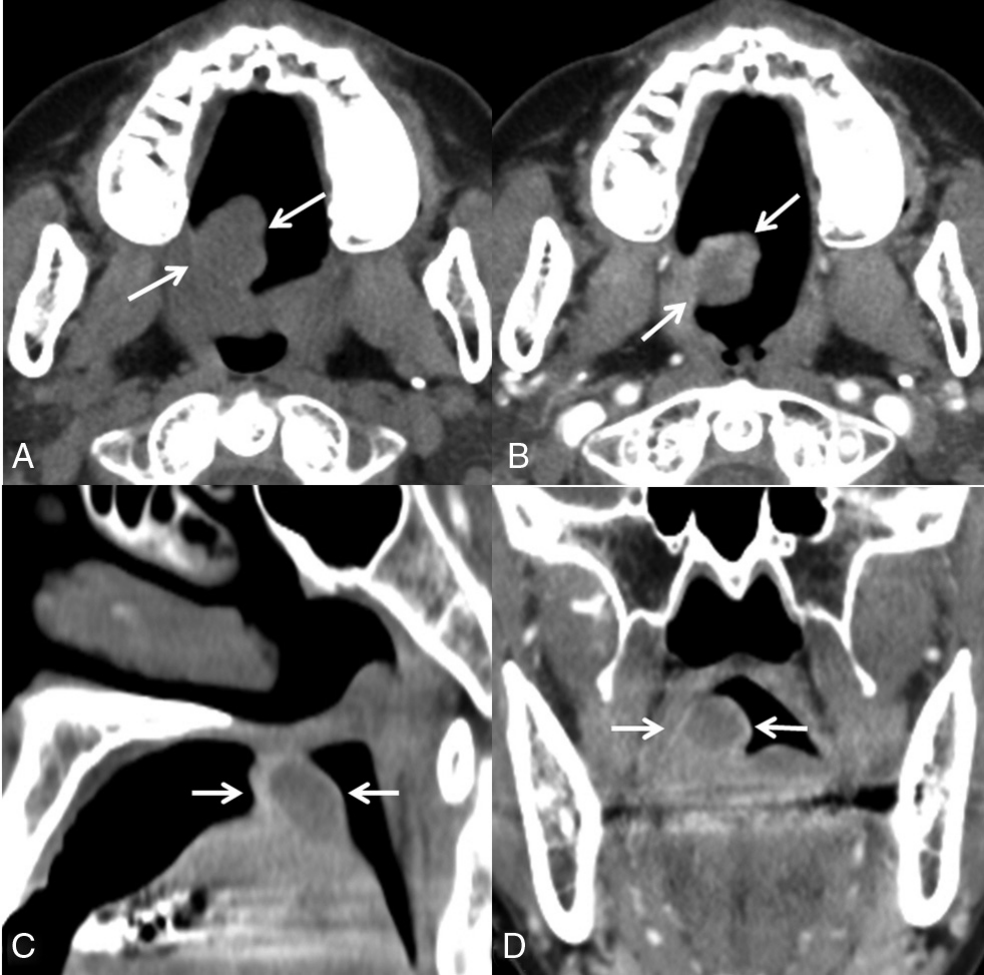
(A) Axial pre-contrast computed tomography (CT) showed a well-delineated homogeneous mass without chondroid matrix calcification. (B–D) Contrast-enhanced CT showing showed a peripheral enhancing mass with internal heterogeneous enhancing portion and the size was measured to be approximately 3.0 cm × 2.0 cm × 1.6 cm. The soft tissue mass was separated from the bone.

**Figure 2:**
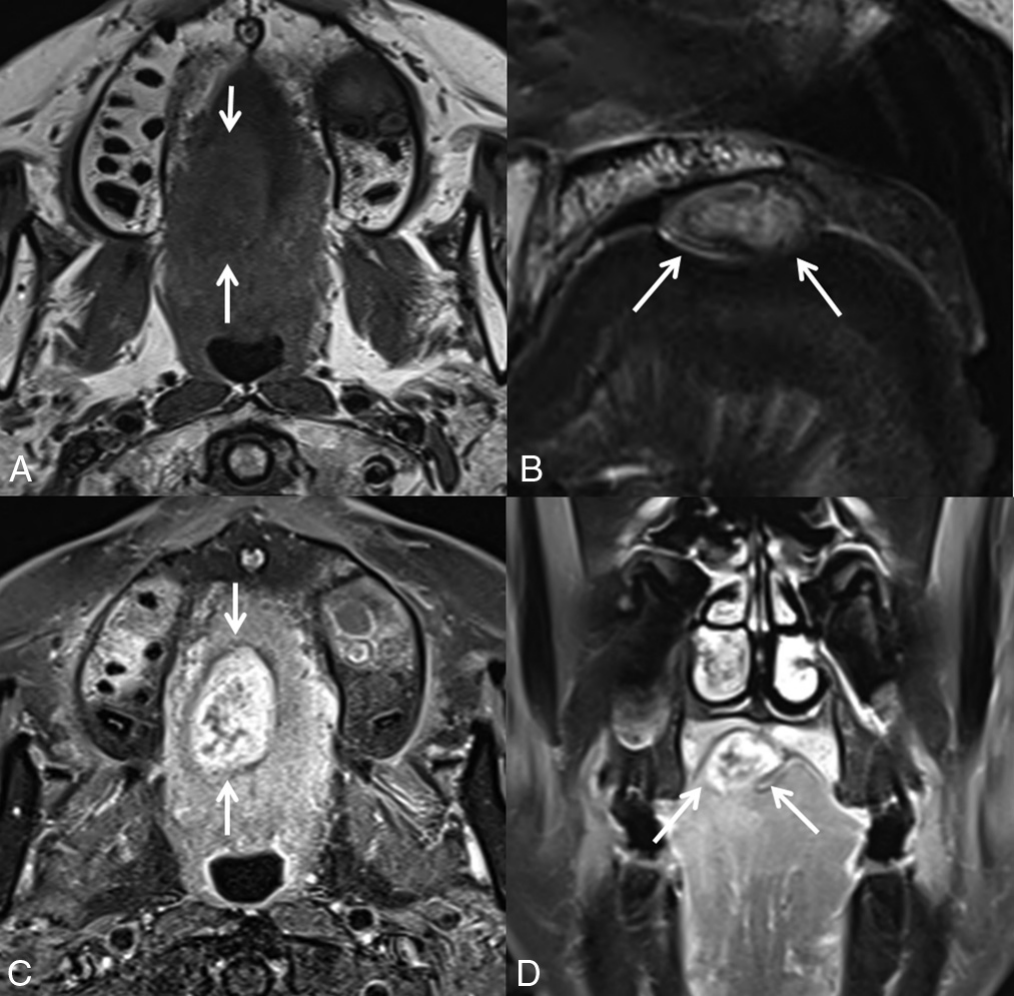
On contrast-enhanced oropharyngeal magnetic resonance imaging (MRI): (A) the oval-shaped lobulated mass with intermediate signal intensity on T1-weighted images, and (B) heterogeneous high signal intensity on T2-weighted images. (C, D) The lobulated mass showed a heterogeneously enhancement crossing the midline.

Radiologically, pre-contrast CT revealed a well-delineated homogeneous soft-tissue mass. Contrast-enhanced CT showed a peripheral enhancing mass with an internal heterogeneous enhancing portion, and the size was measured to be approximately 3.0 cm × 2.0 cm × 1.6 cm. The soft tissue mass was separated from the bone, but there was no chondroid matrix calcification (Fig. 1). On contrast-enhanced oropharyngeal MRI, the oval-shaped lobulated mass showed heterogeneously high signal intensity on T2-weighted images and intermediate signal intensity on T1-weighted images. The lobulated mass showed heterogeneous enhancement, crossing the midline (Fig. 2). These imaging characteristics should be considered when differentiating minor salivary gland benign tumors, STCs, and low-grade chondrosarcomas (LGC).

**Figure 3:**
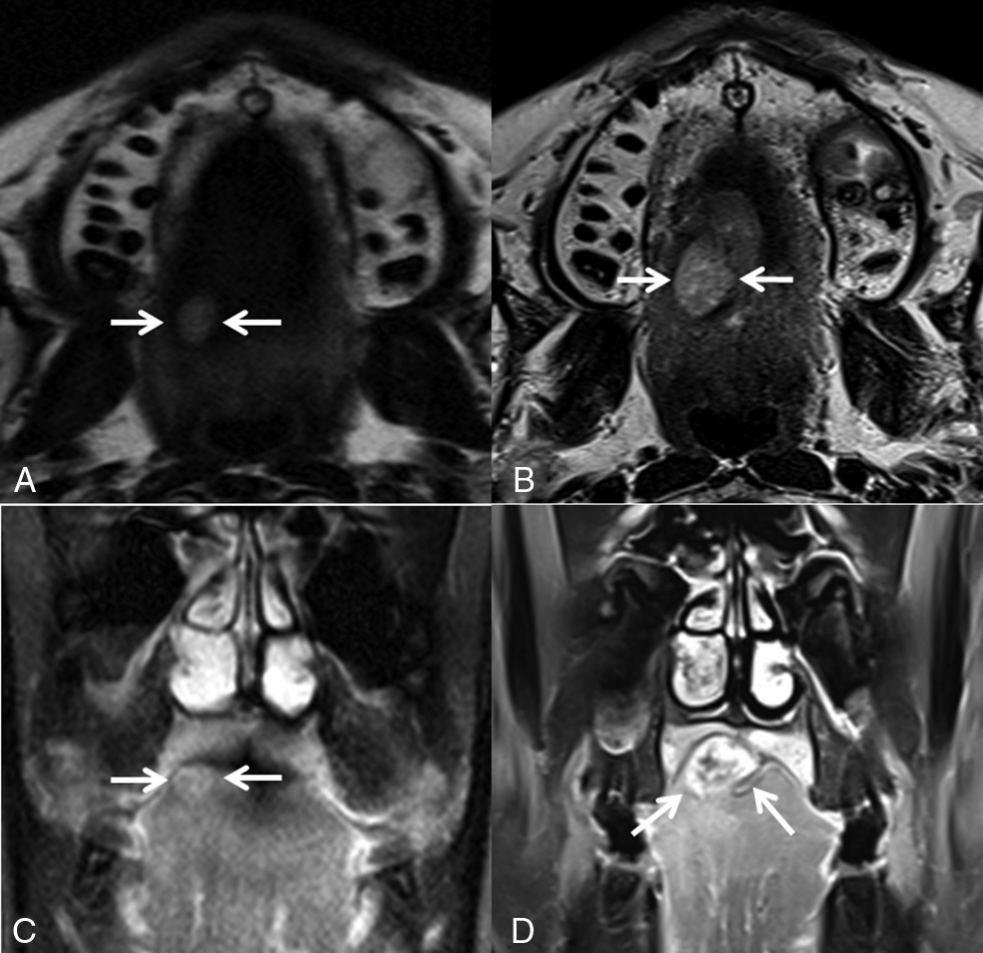
Comparison of previous and present oropharyngeal MRI: (A) the oval-shaped homogeneous mass measuring approximately 1.0 cm × 0.8 cm × 0.8 cm appeared with high signal intensity on a previous T2-weighted image, and (B) the present axial T2-weighted image showing slow-growing 17-year standing lesion with heterogeneous high signal intensity on the right side of the tongue. (C) Previous coronal contrast-enhanced T1-weighted MR image showing homogenously weak enhancement of the mass, and (D) the present MR image showing markedly heterogenous enhancement of the slow-growing mass.

The previous image of 17 years prior showed a soft tissue mass measuring approximately 1.0 cm × 0.8 cm × 0.8 cm appeared with high signal intensity on T2-weighted images and homogeneously weak enhancement. In the same case, at present, the slow-growing lesion showed markedly heterogeneous enhancement and lobulation measuring about 3.0 cm × 2.0 cm × 1.6 cm (Fig. 3).

Intraoperatively, a soft tissue mass was observed attached to the right side of the tongue, crossing the midline. The mass surrounding the normal tissue was excised under general anesthesia.

**Figure 4:**
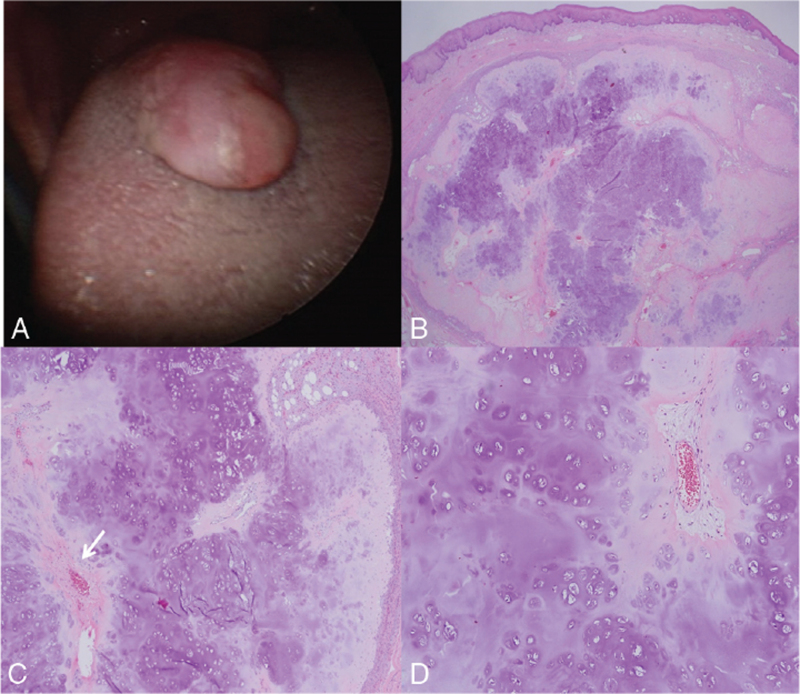
(A) Clinical appearance of a pedunculated mass covered with normal tongue mucosa. (B) Low-magnification view of the soft tissue chondroma in the tongue. It shows a multinodular pattern with large blue areas, constituted of mature hyaline cartilage, surrounded by densely sclerotic fibrous tissue. Hematoxylin-eosin, 1.25×. (C) Irregular located microvasculature (arrow) surrounded by stroma. Hematoxylin-eosin, 40×. (D) The cartilaginous cells are immersed in an extremely abundant matrix. Hematoxylin-eosin, 100×.

Histopathologically, the lesion exhibited a multinodular pattern with slightly well-defined edges. This appearance corresponded to a cartilaginous tumor with multiple nodules composed of mature chondrocytes in an abundant chondroid matrix. The background of this multinodular pattern was intensely sclerotic with some fibroblasts. No signs of infiltration or ulceration of the overlying epithelium were observed (Fig. 4). The histological diagnosis was chondroma, which is a benign lesion. No recurrence was observed during the 1-year follow-up period.

## 3 Discussion

STC develops from embryonal remnants in areas of pre-existing fetal cartilage or where the pluripotent mesenchyme differentiates into cartilage as a result of an irritative stimulus. Typically, tumors are asymptomatic and manifest as slow-growing masses.^[5]^ They are benign cartilage-forming tumors that are usually found near the tendon or joint capsules. The hands and feet were the predominant sites. They consist entirely of mature hyaline cartilage.^[6,7]^ Many patients diagnosed with STC are middle-aged, with ages ranging between 26 and 60 years.^[2,8]^ A slightly higher incidence of female patients has been reported, although a male preponderance has also been reported.^[4]^

The radiological appearance of oral STCs is non-specific. On CT, STCs appear as circumscribed heterogeneously enhancing masses. They frequently demonstrate chondroid calcifications (in 33–70% of cases), which are typically punctate, curvilinear, or ring-like. On MRI, tumors usually have a multilobulated appearance with low-to-intermediate signal intensity on T1-weighted images, high signal intensity on T2-weighted images, and peripheral or septal contrast enhancement. Chondroid matrix calcifications may be evident on MRI as areas of low signal intensity, but these types of calcifications are easily recognized on CT.^[9,10]^ Our case also showed similar findings with a lobulated heterogeneous enhancing mass but no chondroid calcification.

Distinguishing between chondromas and LGC is a common challenge, as the lesions are both histologically and radiographically very similar. Diagnosis is difficult because cartilaginous neoplasms have different histological patterns, ranging from benign chondroid tumors to malignant undifferentiated neoplasms. The MRI features of mesenchymal chondrosarcoma are variable, but the tumor often appears as a lobulated soft tissue mass with low signal intensity on T1-weighted images, variable and heterogeneous signal intensity on T2-weighted images, and complex heterogeneous enhancement after intravenous administration of gadolinium-based contrast material. Chondroid matrix calcifications may be evident on MRI as curvilinear or stippled areas with low signal intensity. Plain radiography and CT are particularly helpful in identifying these calcifications.^[10–12]^ Compared to the MRI image taken 17 years prior, as the size of the lesion increased, there was a change from weakly homogeneous enhancement and clean margins to markedly heterogeneous enhancement and lobulated margins. When we correlated the change in heterogeneous enhancement with pathological findings, it was considered that the sclerotic and fibrotic areas, which are the backgrounds of these nodules, were enhanced, not the multiple nodular areas composed of mature chondrocytes. The distinction between these areas became clear as the size of the lesions increased over time, resulting in markedly heterogeneous enhancement. And the long diameter of the lesion has increased in size from 1 cm to 3 cm over 17 years, and it is thought that it is a slow growing mass, which is considered to have shown as a lobulated margin. Low-grade chondrosarcomas may present variable enhancement patterns and margins, but soft tissue masses with heterogeneous enhancement and lobulated margins are common imaging features in many cases. It can be seen that as the size of tumors increases, low-grade chondrosarcomas have radiologic characteristics.

Radiologically, minor salivary gland tumors, STCs, and LGC were considered differential diagnoses. In our case, it was difficult to make a diagnosis because ring and arc calcifications, which are characteristic findings of chondromas, are not visible. Since the size of a well-defined lesion is less than five–six centimeters and surrounding bone destruction is not clear, it was considered benign rather than malignancy.^[13]^

Surgical excision is the treatment of choice for this condition. Ten to fifteen percent of tumors may recur locally after surgical excision.^[3]^

## 4 Conclusion

STC is a benign cartilage-forming tumor that occurs in the extraarticular soft tissues. Clinically, these tumors present as slow-growing, non-tender masses that are sometimes painful and not attached to the bone. Radiologically, these tumors show well-defined, heterogeneously enhancing masses. Similar to our case, as the size of the tumor increased, it showed LGC characteristics. As the diagnosis of oral STCs may be difficult, radiologists should always keep a differential diagnosis in mind.

## Acknowledgments

This work was supported by a National Research Foundation of Korea (NRF) grant funded by the Korean government (MSIT) (No. 2021-0080).

## Author contributions

**Conceptualization:** Yoonsoo Park.

**Supervision:** Ha Youn Kim, Joo Heon Kim.

**Writing - original draft:** Yoonsoo Park.

**Writing - review & editing:** Dong-Sik Jang, Ha Youn Kim, In-Kyu Yu.
